# Investigation of the Effects of Aquafaba Used Egg Replacers on Pancake Batter and Meringue Foam Rheology and Products Properties

**DOI:** 10.1002/fsn3.72392

**Published:** 2026-10-17

**Authors:** Esra Özyiğit, Selen Erkoç, İpek Özcan, Seher Kumcuoglu, Şebnem Tavman

**Affiliations:** ^1^ Department of Food Engineering, Faculty of Engineering and Natural Sciences Manisa Celal Bayar University Manisa Turkey; ^2^ Department of Food Engineering, Faculty of Engineering Ege University Izmir Turkey

**Keywords:** aquafaba, batter, egg substitute, foam, Large Amplitude Oscillatory Shear (LAOS), vegan

## Abstract

In this study, the effects of adding aquafaba as an egg white substitute on the rheological properties and some quality characteristics (texture, color, SEM) of pancake and meringue samples were investigated. Aquafaba was added to pancake and meringue formulations at different ratios (50%, 100%) instead of egg white. The rheological properties of pancake and meringue batter samples were determined by using the parameters (*η*′_L_ *, η*′_M_, *G*′_L_ *, G*′_M_, *e*_3_, *e*_1_, *v*_3_, *v*_1_) of Large Amplitude Oscillatory Shear (LAOS) analysis. The Lissajous curves showed that substituting aquafaba for egg white reduces stress values. The structure of samples was affected by aquafaba; dissipated and stored energy were decreased as a result. According to LAOS parameters, the nonlinear behavior of the pancake and meringue samples was strain stiffening. The properties of pancake and meringue samples were shear thinning and shear thickening from the viscous perspective, respectively. It was found that the hardness values of pancakes and meringue samples decreased by using aquafaba (*p* < 0.05). According to SEM, the porosity of the pancake samples decreased, and the meringue samples showed a more layered and fragile structure with the increase in the substitution ratio.

## Graphical abstract

Aquafaba was evaluated as a plant‐based egg‐white replacer in meringue and pancake systems. The effects on the quality of the products and their structural properties were evaluated by density, texture, color, SEM, and nonlinear rheological (LAOS) analyses. Aquafaba‐based products may offer a potential alternative for consumers avoiding eggs due to vegan dietary preferences or egg allergies.

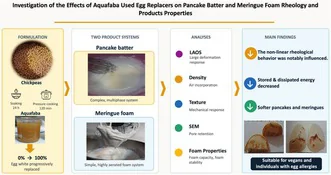

**Abbreviations**
LAOS: large amplitude oscillatory shear
M100: meringue sample containing 100% aquafaba
M50: meringue sample containing 50% aquafaba
MC: meringue control
P100: pancake sample containing 100% aquafaba
P50: pancake sample containing 50% aquafaba
PC: pancake control

## 1 Introduction

Egg is one of the basic ingredients frequently used in food applications due to its various functional properties such as foaming, gelling, thickening, emulsifying, and coloring (Uysal et al. 2019). However, the various health risks associated with egg consumption (cardiovascular diseases, egg allergy) or the increasing popularity of consumers' vegan and vegetarian diet preferences and more food product options lead to the search for alternative sources that can be used instead of eggs (Agrahar‐Murugkar et al. 2016). Although there is increasing interest in egg alternatives, these alternatives are expected to preserve the sensory and functional properties that eggs typically provide (Mustafa et al. 2018). Over the years, different substitutes have been tried to replace eggs partially or completely. Plant proteins such as pea, soy, and lupine protein and animal proteins (hydrolysates, collagen, whey protein concentrate, gelatin, etc.), wheat starch, various hydrocolloids and emulsifiers, fruits and vegetables, oily seeds (chia, lintseed, rapeseed) are used as egg substitutes (Agrahar‐Murugkar et al. 2016). Chickpea, which is one of the important vegetable protein sources, is a legume that is widely consumed in the World, contains high‐quality protein, and therefore has important nutritional properties (El‐Adawy 2002). The term “aquafaba” comes from the Latin “water” (aqua) and “beans” (faba) and refers to both the boiling water of the legumes and the liquid in canned legumes (Buhl et al. 2019). Aquafaba turns into white foam when whipped up, and is used in food applications as a fresh liquid ingredient or as a freeze‐dried powder ingredient to increase functional properties (Mustafa et al. 2018). Aquafaba contains significant amounts of carbohydrates (predominantly starch), protein, saponin, cellulose, pectin, and viscosity‐increasing insoluble fibers, highly soluble fibers (Stantiall et al. 2018). Foaming, emulsifying, and thickening are some functional properties of aquafaba. Aquafaba as an egg substitute is an interesting subject due to the functional features it provides and its vegetable origin (Mustafa et al. 2018). The usage of aquafaba as an egg substitute in the formulations of vegan mayonnaise (Ozcan et al. 2023), cookie (Aksu and Seçim 2025), meringue (Stantiall et al. 2018), sponge cake (Mustafa et al. 2018), cake (Aslan and Ertaş 2020), meringue and mayonnaise (Lafarga et al. 2019), mousse (Damian et al. 2018), oat‐based vegan yogurt (Raikos et al. 2020) has been the subject of many studies.

In this study, meringues are considered ideal model systems for examining the behavior of egg substitute foams, which form the basis of bakery products, due to their simple composition (Licciardello et al. 2012). Meringue is a confectionery product made from sugar and whipped egg whites. It is generally obtained by whipping the egg white into foam and then cooking it at a certain temperature and time after adding sugar to the foam formed. Pancake samples, on the other hand, are examined in terms of the use of aquafaba in egg substitute studies because they represent more complex, fluid emulsion/suspension systems with a continuous aqueous phase of starch granules, proteins, and lipids (Chesterton et al. 2013; Hesso et al. 2015). The exact ingredients may vary depending on the formulation, as egg‐free pancakes have also been developed using aquafaba and other plant‐based egg replacers (Bianchi and Simonato 2025; Tan et al. 2025). Although it is known for different recipes in different countries, it is consumed as Crepe in France, Hotcake in Korea, and Doriyaki in Japan. A light and fluffy texture is observed in the pancake, which has a juicy dough structure (Cho et al. 2019). Egg white foaming is an important criterion (O'Charoen et al. 2014), and thus, the quality and rheological properties of meringue and pancake samples are affected by replacing egg white with aquafaba. The focus of the rheological characterization of meringue and pancake samples has been the small strain amplitude tests (SAOS) in the linear viscoelastic region (LVR). The rheological properties are limited in the LVR by evaluating the initial stress responses of the sample in SAOS. Thus, large amplitude oscillatory analysis (LAOS) is utilized to characterize the rheological properties of samples in the nonlinear region in more depth (Hyun et al. 2011). As far as we know, there is no study on the investigation of the nonlinear rheological characteristics of pancake and meringue samples when aquafaba is used in the recipe.

Aquafaba is not a single component foaming agent, but a complex mixture of soluble proteins, saponins and carbohydrates (Buhl et al. 2019; Tufaro and Cappa 2023; Tan et al. 2025). Proteins and saponins form interfacial films, while soluble carbohydrates and polysaccharides influence the properties of the continuous phase (Laursen et al. 2025). The combination of such components leads to a heterogeneous and strain‐dependent structure that cannot be characterized by linear rheological parameters alone (Erturk et al. 2023). For this reason, LAOS analysis was selected to quantify intracycle elastic and viscous responses, energy storage and dissipation, and structural evolution during large deformations (Ptaszek 2014; Erturk et al. 2023). In this study, aquafaba is used as an egg white substitute to prepare meringue and pancakes that can be consumed by individuals who do not consume eggs for various reasons, as well as to examine the results of the utilization of aquafaba as an egg replacement on batter rheology and meringue and pancake quality.

## 2 Materials and Methods

### 2.1 Materials

Sugar, salt, baking powder, wheat flour, and eggs were supplied from the local market to make pancakes. Also, eggs and powdered sugar were purchased from the local market for the meringues. Chickpeas ( *Cicer arietinum* L., Koçbaşı type) were provided from the local market (Arbel Legumes and Grains, Mersin, Turkey) to produce aquafaba.

### 2.2 Preparation of Aquafaba

Chickpea cooking water was prepared using the method specified by Lafarga et al. (2019). Accordingly, chickpea grains were first kept in tap water for 24 h at a chickpea‐to‐water ratio of 1:3 (w/v). At the end of 24 h, water and chickpeas were separated, and water was discarded. Chickpeas kept in water were cooked for 120 min in a pressure cooker (Schafer, Galaxis) with a chickpea‐to‐water ratio of 1:1.5 (w/v). After cooking, the chickpea and aquafaba are separated. The pH value of the separated aquafaba (pH: 6.15 ± 0.01) was set to approximately 5 with the help of lemon juice and stored at 4°C.

### 2.3 Production of Pancake and Meringue Samples

The pancake samples were prepared as stated by the modified method designated by Shih et al. (2006). Aquafaba (50%, 100%) was added to the formulation separately, replacing egg white. Pancake control (PC) is the sample containing only egg white. The formulations of the pancake samples are given in Table 1. All dry ingredients were mixed to prepare the pancake batter. After mixing aquafaba and water in a separate bowl, dry ingredients were added to liquid ingredients and mixed with a spatula for 45 s. After mixing, 40 g of dough was cooked for 3 min in a pre‐oiled and heated pan, then turned over and cooked for another 3 min.

**TABLE 1:** Formulation of pancake and meringue samples.

Ingredients	Pancake samples		
	PC	P50	P100
Wheat flour (g)	100	100	100
Egg white (g)	40	20	0
Aquafaba (g)	0	20	40
Sugar (g)	20	20	20
Salt (g)	2	2	2
Baking powder (g)	4.5	4.5	4.5
Water (g)	108.5	108.5	108.5

Ingredients	Meringue samples		
	MC	M50	M100
Powdered sugar (g)	250	250	250
Egg white (g)	250	125	0
Aquafaba (g)	0	125	250

Abbreviations: M100: meringue sample containing 100% aquafaba; M50: meringue sample containing 50% aquafaba; MC: meringue control sample; P100: pancake sample containing 100% aquafaba; P50: pancake sample containing 50% aquafaba; PC: pancake control sample.

The meringues were prepared by modifying the classical meringue preparation method given in Gisslen (2011). Aquafaba (50%, 100%) was added to the formulation separately, replacing egg white. A meringue control (MC) was made from only egg white. The formulations of the meringue samples are given in Table 1. While preparing the meringue foam, 250 g of egg white and aquafaba were whisked with the KitchenAid Classic Mixer (KitchenAid, St. Joseph, MI, USA), at speed 5 (160 rpm) for 3 min, then whisked at speed 10 (280 rpm) for 6 min. Then, 250 g of powdered sugar was added gradually while whisking was in progress, and whisking was continued for 6 min, including the time during which the powdered sugar was added. After the whisking process was completed, the foams with a diameter of 3 cm were placed on trays and baked in an oven (Vestel, Turkey) at 110°C for 90 min. After cooking, the meringues were left to cool for 1 h at room temperature and stored in polyethylene bags at room temperature to wait until analysis.

### 2.4 Analysis of Aquafaba and Egg White

#### 2.4.1 Determination of Protein Concentration, Total Solid Content, and pH

The protein content of egg white and aquafaba was calculated using the FP‐528 Nitrogen/Protein Analyzer (Leco, St. Joseph, USA). Before analysis, samples were dried with Alpha 1‐2 LD plus Lyophilizer (Martin Christ, Göttingen, Germany) at −56°C for 48 h. The concentration of total solids was examined until a constant weight was found using a drying oven (Memmert, Türkiye) functioning at 105°C (Grossi Bovi Karatay et al. 2022). The pH was determined in a pH meter (HI 2211, HANNA Instruments).

#### 2.4.2 Determination of Foaming Capacity (FC) and Foam Stability (FS)

The FC and FS of aquafaba and egg white were calculated by using the method specified by Mustafa et al. (2018). According to this, 5 mL of egg white or aquafaba was diluted with 10 mL of water in a 150 mL graduate. After that, it was whisked using the KitchenAid Classic Mixer (Kitchen Aid, St. Joseph's, MI, USA) for 3 min with speed 10 (280 rpm) in a stationary bowl (4.3 L). *V*_sample_ represents the initial volume of undiluted aquafaba or egg white used in the analysis. The volumes of the foams of the whisked samples were measured at the 0th minute (*V*_F0_) and the 30th minute (*V*_F30_). FC and FS were determined using Equations (1) and (2), respectively.

(1) $$\mathrm{FC}\;\left(\%\right)=\frac{V_{F0}}{V_{\text{sample}}}\times 100$$

(2) $$\mathrm{FS}\;\left(\%\right)=\frac{V_{F30}}{V_{F0}}\times 100$$

### 2.5 Analysis of Meringue and Pancake Batter

#### 2.5.1 Density

The densities of the pancake and meringue batters were measured using an Elcometer (1800, Elcometer, Manchester, UK) pycnometer. The pycnometer with a volume of 100 mL was weighed empty for calibration purposes before the analysis and then the pycnometer was filled with batter samples. The excess samples were removed and then the obtained value was recorded. The total mass of the samples was divided into their volumes to determine density values (Gularte et al. 2012).

#### 2.5.2 Rheological Properties

The nonlinear rheological behavior of pancake and meringue samples was determined with a TA DHR‐3 (TA Instruments Inc., New Castle, DE, USA) Rheometer by using a parallel plate (40 mm diameter) with a gap height of 2 mm. The measurements were made in triplicate at 25°C and between the strain values 0.01% and 200% at a constant frequency of 1 Hz (6.28 rad/s). The nonlinear rheological parameters of samples were determined using elastic modulus, viscous modulus, Chebyshev coefficients, dynamic viscosity, and Lissajous curves (Ozcan et al. 2023).

### 2.6 Analysis of Meringue and Pancake Samples

#### 2.6.1 Texture

The texture of the samples was measured 24 h after cooking with the TA‐XT2i Texture Analyzer (StableMicrosystems, Surrey, UK). Texture analysis of pancake samples was applied by modifying the method specified by Fajardo and Ross (2015). For this purpose, a 25 mm probe was applied with a constant speed (2 mm/s), trigger force of 50 g, and 50% tension. The texture analysis of the meringue samples was carried out by modifying the method specified by Stantiall et al. (2018). A single compression was applied to the meringue samples up to the breaking point. For this purpose, a trigger force of 5 g was used with a 35 mm probe with a velocity of 1.0, 1.7, and 10 mm/s for the pretest, test, and posttest, respectively.

#### 2.6.2 Color

The surface color and crumb colors of the pancake samples and the color of the ground and homogenized meringue samples were determined using the CM‐400d Minolta Color Reader (Konica Minolta, Osaka, Japan). The effect of using different ratios of aquafaba in different samples was determined by measuring L* (brightness), a* (+ red, − green), b* (+ yellow, − blue) color values.

#### 2.6.3 Scanning Electron Microscopy (SEM)

The microstructure of the produced samples was visualized with SEM. The cooked samples were cut into cubes of 1 ${\mathrm{cm}}^{3},$ volume from the inner sides, dried with Alpha 1‐2 LD plus Lyophilizer (Martin Christ, Göttingen, Germany) at −56°C for 24 h, and covered with gold. SEM analysis was performed with an Apreo S scanning electron microscope (Thermo Scientific, Waltham, USA). Images were recorded at ×1000 magnification.

### 2.7 Statistical Analysis

Experimentally obtained data were statistically evaluated using one‐way analysis of variance (ANOVA) to find out whether there was a significant difference (*p* < 0.05) between the samples made with different formulations. When a significant difference was found, the comparison was made by using Tukey's multiple comparison tests. All statistical analyses were performed using IBM SPSS Statistics 20 (IBM, Chicago, USA). All formulations were prepared in three independent production batches. Aquafaba extraction and the preparation of pancake and meringue samples were carried out separately for each batch. Each analysis was performed in triplicate for every independent production batch. Therefore, the experimental design comprised three independent production replicates (*n* = 3) and three technical replicates per production replicate.

## 3 Results and Discussion

### 3.1 Results of Aquafaba and Egg White

#### 3.1.1 Protein Content, Total Solid Content, pH

The protein content of aquafaba was found 17.42% in dry matter and 1.12% on a wet basis (Table 2). This result is supported by the literature that the protein content of aquafaba varies from 0.7 g/100 g to 1.5 g/100 g. Aquafaba composition is affected by the extraction conditions, chickpea cultivar and type, composition, and structure of the seed and cell wall (He et al. 2021). Hence, the protein content of aquafaba was affected by the chickpea genotype and preparation conditions such as soaking and cooking time. Alsalman et al. (2020) indicated that longer cooking times led to an increase in the protein content of aquafaba in their study. They found that the protein content of liquid aquafaba samples ranged from 0.5% to 1%. The protein concentration in egg whites has been determined to be 73.002% on a dry weight basis and 9.24% on a weight basis. The total protein concentration in egg whites is reported to range from 10% to 11% (Chang et al. 2018; Sarantidi et al. 2023).

**TABLE 2:** The protein, foaming capacity (FC), and foam stability (FS) values of aquafaba and egg white.

Sample	Protein (%) (dry basis)	FC (%)	FS (%)
Egg white	73.002 ± 0.5^b^	781.66 ± 53.9^b^	97.22 ± 0.3^b^
Aquafaba	17.42 ± 0.62^a^	326.66 ± 29.4^a^	84.03 ± 2.5^a^

*Note:* Different letters in the same column (a, b, c) indicate significant differences between means (*p* < 0.05).

The total solid content of aquafaba generally ranges from approximately 5% to 8% (He et al. 2021). For instance, total solid contents of approximately 4.9%–7.0% have been reported for 10 different commercial chickpea waters (Shim et al. 2018). Consistent with the literature, the total solid content was determined to be 6.42%. Previous studies have indicated that the total solid content of egg white is approximately 12% (Chang et al. 2018). In agreement with these findings, the total solid content of the egg white used in the present study was determined to be 12.69%.

The pH of aquafaba was 6.15 ± 0.01, which was consistent with the slightly acidic values of 6.15, 6.26 previously reported for chickpea aquafaba (Stantiall et al. 2018; Tufaro and Cappa 2023). Buhl et al. (2019) and Lafarga et al. (2019) reported that aquafaba FS increased at pH values close to the isoelectric point (pH 4.6). Accordingly, the pH of aquafaba was adjusted to 5 before its use in the formulations.

#### 3.1.2 FC and FS

The FC of egg white (781.66%) was found much higher than aquafaba (326.66%) (Table 2). On the other hand, the foaming stability for the egg white foam and aquafaba is 97.22% and 84.03%, respectively (Table 2). Similarly, Stantiall et al. (2018) and Buhl et al. (2019) found that fresh egg white foam capacity is significantly higher than foam prepared from the aquafaba. Stantiall et al. (2018) stated that there is a strong relationship between protein content and FC. So, the lower protein content of aquafaba than egg white could explain this result. Mustafa et al. (2018) indicated that differences in the aquafaba composition and shorter whipping time also affect the FC of aquafaba.

There are several factors that affect a solution's foaming ability, including surface activity, surface tension, the film‐forming properties of the foaming agent, and rapid absorption capability on air–liquid interfaces (Liu et al. 2009). In foam formation, proteins play an important role because they are amphiphilic, having hydrophilic ends attracted to water and hydrophobic ends attracted to air (Mariotti et al. 2013). Because of its high albumin content, egg white is a common foaming agent in bakery and confectionery foods, providing stable foams. On the other hand, aquafaba contains varying quantities of carbohydrates, proteins, and saponins, which contribute to its foaming or gelling properties. Pulses also contain a considerable amount of albumin, and this may be the reason for the stable foams like egg white (Stantiall et al. 2018). In addition, saponins can interact with proteins to form high‐molecular‐weight complexes. According to the literature, the saponin content of chickpea seeds ranges from approximately 15.30 to 40.92 mg/g (Kaur et al. 2016). It has been reported that soaking reduces the saponin content by 44.94%, while chickpea cooking water contains 4.5 mg/g of saponins (Stantiall et al. 2018). This causes protein aggregation and alters their thermal stability and surface characteristics when subjected to heat (He et al. 2021). These interactions may ultimately enhance the foaming and emulsifying properties of proteins. Similar results were found with the results of Mustafa et al. (2018), who report the FC and foaming ability of 182%–476% and 77%–92%, respectively. FC and ability differences are directly related to chickpea varieties, composition, and preparation of aquafaba conditions (Shim et al. 2018). Additionally, Kargar and Sourki (2025) reported that the foaming ability of microwave‐assisted aquafaba was 276.66%, while the FS of conventionally extracted aquafaba was 50.00%.

### 3.2 Results of Meringue and Pancake Batter

#### 3.2.1 Density

Density values of pancake and meringue batter with different concentrations of aquafaba are 1.049–1.123 and 0.123–0.156 g/mL, respectively (Table 3) The highest batter density values were obtained from both pancake and MC samples. As the ratio of aquafaba substitution increased, the pancake and meringue batter density decreased. This indicates that the batters made with aquafaba have a higher amount of incorporated air and are lighter. The density values of cake samples decreased as the aquafaba substitution ratio increased, according to Mustafa et al. (2018). The results are consistent with those of Aslan and Ertaş (2020), who utilized aquafaba as an egg substitute. They reported a decrease in density values due to the use of aquafaba. Alavi et al. (2020) found the density value of a meringue batter sample made from egg white protein powder was 0.61 g/mL (Shim et al. 2018). In addition, Kargar and Sourki (2025) reported that the density of meringue batter, which contained varying amounts of microwave‐assisted extracted aquafaba, ranged from 0.32 to 0.49 g/mL. Although lower batter densities were achieved relative to those reported in previous studies, Campbell and Mougeot (1999) defined the typical density of egg white meringues as approximately 0.18 g/mL. Laursen et al. (2025) indicated that variations in the density values of meringue samples might arise from differences in the recipe, the amount of sugar used, and the mixing procedure.

**TABLE 3:** Density values of meringue foam and pancake dough with different concentration aquafaba.

Sample	Density (g/mL)	Sample	Density (g/mL)
PC	1.123 ± 0.003^c^	MC	0.156 ± 0.001^c^
P50	1.077 ± 0.03^b^	M50	0.138 ± 0.002^b^
P100	1.049 ± 0.03^a^	M100	0.123 ± 0.001^a^

*Note:* Different letters in the same column (a, b, c) indicate significant differences between means (*p* < 0.05).

#### 3.2.2 Rheological Properties of Meringue and Pancake Samples

##### 3.2.2.1 Determination of the Nonlinear Viscoelastic Region by Strain Sweep Test

The elastic modulus (*G*′), and the viscous modulus (*G*″) are used to examine strain sweep tests, and results are given in Figure 1. For all pancake and meringue samples, *G*′ values are greater than *G*″ values. This indicated that the behavior of samples was more elastic and solid‐like than viscous in the LVR. The highest *G*′ and *G*″ values were obtained with control samples for pancakes and meringues and the crossover points for *G*′ and *G*″ was not affected by the addition of aquafaba. Pancake batters showed typical Type I behavior where the applied strain results in deformation and destroys the structure defined by strain thinning (Hyun et al. 2011). This type of behavior occurs because of the microstructure alignment with flow direction and the breaking up of agglomerated particles into smaller ones, both of which increase fluidity (Hyun et al. 2011). The *G*′ and *G″* values for pancake samples varied between 0–200 and 0–100 Pa, respectively. Parallel to these findings, Lorente‐Bailo et al. (2021) reported that the *G*′ and *G*″ values of pancakes made from different flours ranged between 5.50–279.13 and 3.51–75.75 Pa, respectively.

**FIGURE 1:**
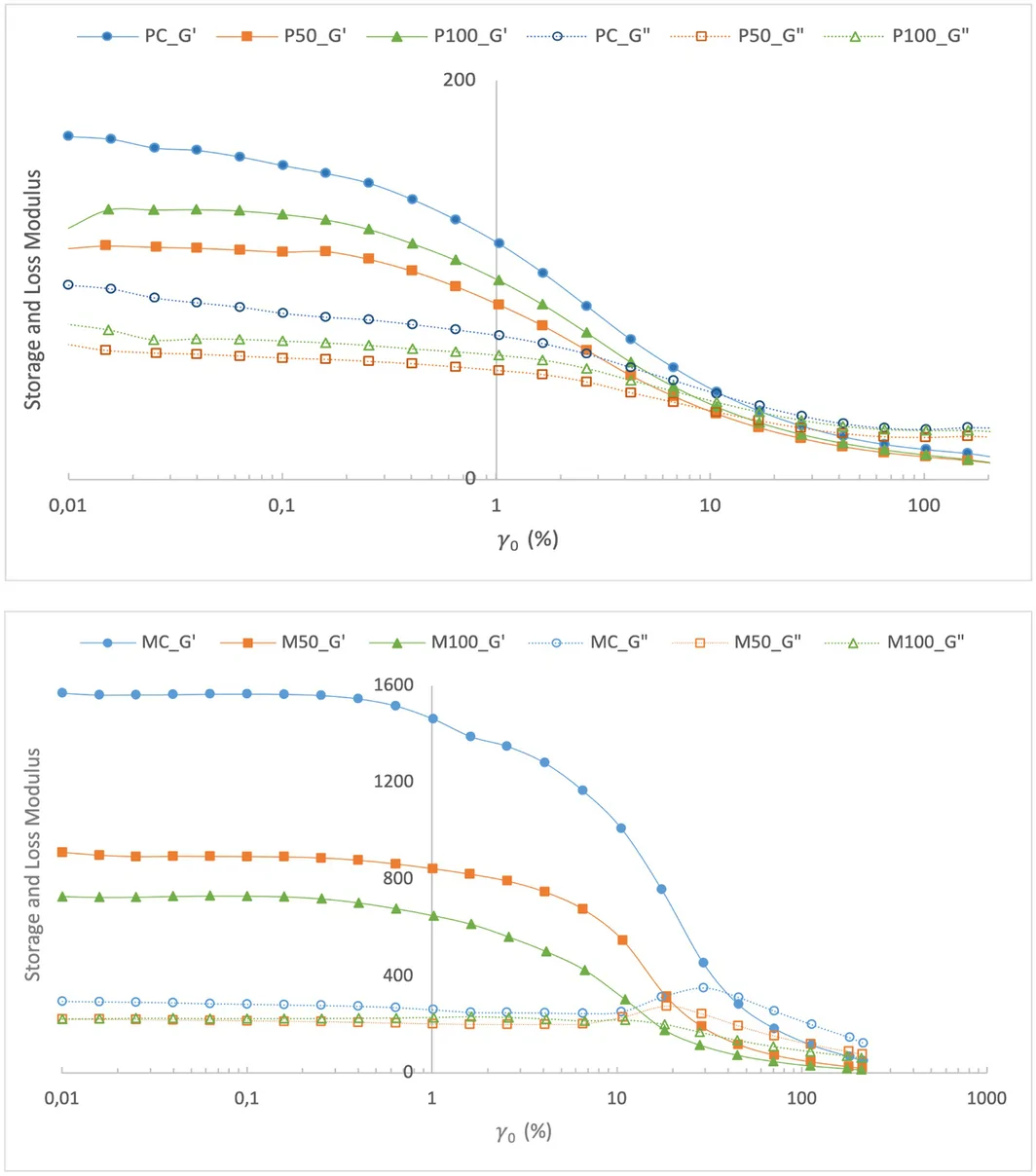
Strain sweep plots of pancake and meringue as a function of aquafaba level (0%, 50%, 100%) at 6.28 rad/s.

*G*′ values showed a significant decrease with the addition of aquafaba for meringue samples. *G*′ values varied between 0 and 1600 Pa and *G*″ values ranged between 0 and 400 Pa. The nonlinearity begins with ~0.5%–1% strain for all meringue samples at 6.28 rad/s. On the other hand, the use of aquafaba shifted the *G*′*–G*″ crossover point to lower strain values. *G*′ is much greater than *G*″ samples, which is typical of weak physical gels. On the other hand, increasing strain amplitude caused a decrease in *G*′ and an increase in *G*″, then *G*″ decreased at higher strain values. This type of behavior is described by weak strain overshoot, which is Type III (Hyun et al. 2011). The irreversible deformation and flow are observed because of the microstructural deformations at higher strain values in this type of behavior (Anvari and Joyner (Melito) 2018). Similar findings were found by Nastaj et al. (2021) for meringue samples that were obtained from whey protein isolate. He et al. (2021) stated that aquafaba generates a weak hydrogel due to the presence of water‐binding proteins, water‐soluble carbohydrates, and insoluble fiber, which predominantly includes cellulose and pectin, along with their interactions.

##### 3.2.2.2 The Evaluation of General Lissajous Curves

The Lissajous curves, which are closed‐loop plots of stress vs. strain or stress versus rate of strain, provide a graphical representation of samples' behavior like strain softening/hardening and shear thickening/thinning (Hyun et al. 2011). Figure 2 shows that bigger stress responses (black lines) are obtained by increasing strain amplitude for all samples. At low strains, linear viscoelastic properties are defined by the relatively small elliptic shape in the middle of the figures. The rotation and the shape of the loops are changed by the increasing strain where the nonlinear region begins, and it is more distinctive for meringue samples. The materials' elastic perspectives are defined by the stress–strain plane (green lines), and the area of these curves is used to evaluate stored energy. The viscous properties of the material are defined by the projection in the stress‐shear rate plane (red lines) and the dissipated energy can be analyzed by the area of these curves (Duvarci et al. 2017). As shown in Figure 2, aquafaba substitution reduced the dissipated and stored energies in both pancake and meringue samples. In addition, this is more distinctive for the meringue samples, especially for the meringue containing 100% aquafaba. When the viscous projection of meringue samples is examined, increasing strain causes clockwise rotation, and stated the behavior of intracycle shear thinning (Ewoldt et al. 2008). The M100 sample showed clockwise rotation at smaller strain values compared to MC and M50. The lowest stress values were obtained at 50% and 100% substitutions of aquafaba for pancake and meringue samples, respectively.

**FIGURE 2:**
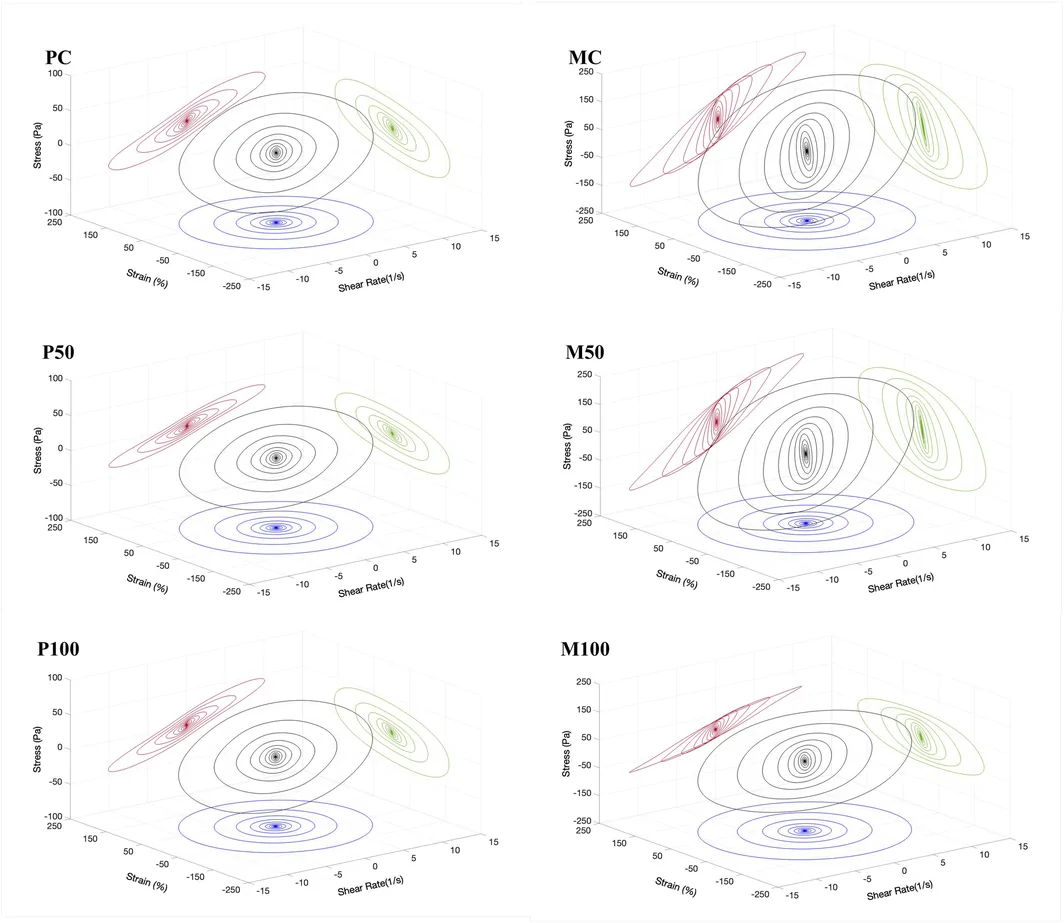
The unnormalized 3D Lissajous–Bowditch curves for pancake and meringue samples (stress response: black lines, elastic component: green lines, viscous component: red lines).

##### 3.2.2.3 The Evaluation of Lissajous–Bowditch Curves From Elastic and Viscous Perspective

The Lissajous‐Bowditch curves are plotted as elastic and viscous parts of the material by normalization, and so the nonlinear characteristics of the material can be more easily distinguishable (Ewoldt et al. 2008). The elastic Lissajous‐Bowditch curves define the material's elastic properties, and normalized stress ($\sigma /\sigma_{\max}$) with normalized elastic stress ($\sigma^{\prime }/\sigma_{\max}^{\prime }$) are presented against the normalized strain values $\left(\gamma /\gamma_{\max}\right)$. In contrast, the viscous part of the material is defined by the curves presented in the form of normalized stress ($\sigma /\sigma_{\max}$) and normalized viscous stress $\left(\sigma^{\prime \prime }/\sigma_{\max}\right)$ versus normalized strain rate values $(\dot{\gamma }/\dot{\gamma_{\max})}$.

The LVR for the elastic and viscous stress curves is described by the inner and outer loops, respectively. In contrast, the nonlinear region is defined as the opposite of the LVR for the elastic and viscous curves (Yazar et al. 2017). As shown in the graphs (Figure 3), purely elliptical and circular shapes indicate a LVR in the stress–strain and stress–strain rate planes, respectively. Experimental noise affected Lissajous curves for P50 and P100, causing wavy structures. The stress loops became wider with the addition of aquafaba and increased strain amplitude for both the pancake and meringue samples in the stress–strain plane, showing the effect of aquafaba on sample microstructure (Figure 3). These wider loops indicate that higher energy consumption occurs during flow and, as a result, more viscous behavior, implying irreversible breakdown of structure (Yazar et al. 2017). It is more obvious for meringue samples; the stress loops became wider at lower strain values for M100 samples. In addition, distortion from the elliptical shape reveals the material's nonlinearity (Ewoldt et al. 2008). The network structure of pancake samples tends to stretch in one direction during the oscillatory flow and stretch again in the opposite way during flow, resulting in strain hardening (Duvarci et al. 2017).

**FIGURE 3:**
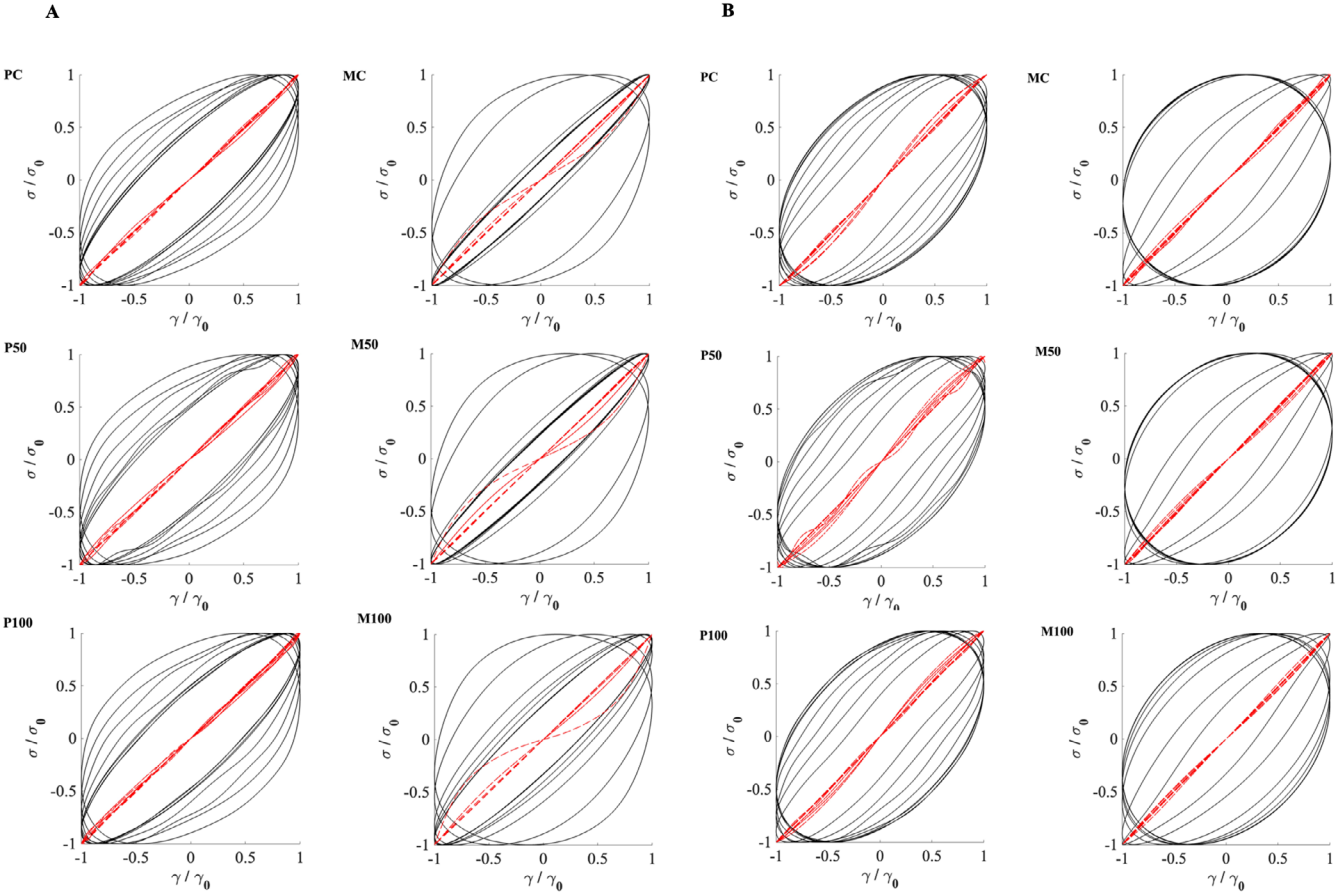
(A) The normalized elastic Lissajous–Bowditch curves for pancake and meringue samples; (B) the normalized viscous Lissajous–Bowditch curves for pancake and meringue samples.

In contrast, the viscous stress loops became narrower with increasing strain amplitude and aquafaba addition in both the pancake and meringue samples. This indicates that M100 exhibited fluid‐like behavior with narrower ellipses than other meringue samples. As can be seen in the graph (Figure 3), the nonlinearity is more distinguishable from the elastic perspective. Both elastic and viscous Lissajous curves complement one another and provide more detailed information about nonlinearity.

##### 3.2.2.4 Elastic and Viscous LAOS Parameters

*G*′_1_ and *G*″_1_ are the parameters of the first harmonic data and show the viscoelastic responses in the LVR. Ewoldt et al. (2008) stated that these first harmonic data were unable to distinctly define viscoelastic responses at higher strain and described new moduli by using Fourier transform for the non‐LVR. According to Ewoldt et al. (2008), the elastic modulus is defined by the minimum strain modulus (*G*′_M_) and the large strain modulus (*G*′_L_). The slope of the tangent at the lowest strain (𝛾 = 0) and the slope of the secant at the highest strain (𝛾 = 𝛾_0_) define *G*′_M_ and *G*′_L_, respectively. In the LVR, *G*′_*1*,_ *G*′_*M*,_ and *G*′_*L*_ are equal, and Lissajous‐Bowditch curves have an elliptical shape. In addition, viscous properties are defined by the dynamic viscosity which is large‐rate dynamic viscosity (*η*′_L_) and minimum‐rate dynamic viscosity (*η*′_M_). *η*′_L_ and *η*′_M_ are determined by the viscosities at the largest (𝛾 = ±𝛾_0_) and smallest shear rates (𝛾 = 0), respectively. Melito et al. (2012), interpreted these parameters to define nonlinear elastic and viscous behaviors of material. The ratios of *G*′_L_ to *G*′_M_ and *η*′_L_ to *η*′_M_ are used to interpret nonlinearity. The strain softening behavior is indicated by *G*′_L_/*G*′_M_ < 1, while the strain stiffening behavior is indicated by *G*′_L_/*G*′_M_ > 1. Additionally, the shear thickening and thinning properties are also defined with the same intervals, according to *η*′_L_/*η*′_M_, respectively. Figure 4 show that the ratio of *G*′_L_ to *G*′_M_ is equal to 1 in the LVR, but the ratio becomes different at the nonLVR. The pancake samples showed strain softening (*G*′_L/_ *G*′_M_ < 1) between 2% and 10% strain followed by strain stiffening behavior (*G*′_L/_ *G*′_M_ > 1) up to 200% strain value in the nonlinear region (Figure 4). The *G*′_L/_ *G*′_M_ value decreased as the strain amplitude increased, and the samples' behavior changed to strain softening at 200% strain. Figure 4 demonstrates that *η*′_L_/*η*′_M_ values are equal to 1 in the linear region and indicate shear thinning behavior after 2% strain value in the nonlinear region. Meringue samples exhibit strain stiffening (*G*′_L/_ *G*′_M_ > 1) and shear thickening (*η*′_L_/*η*′_M_ > 1) in the nonlinear region. But then, samples exhibited shear thinning behavior (*η*′_L_ */η*′_M_ < 1) at higher strain values (200%). The microstructural deformations at higher strain values may be the reason for this behavior for meringue samples.

**FIGURE 4:**
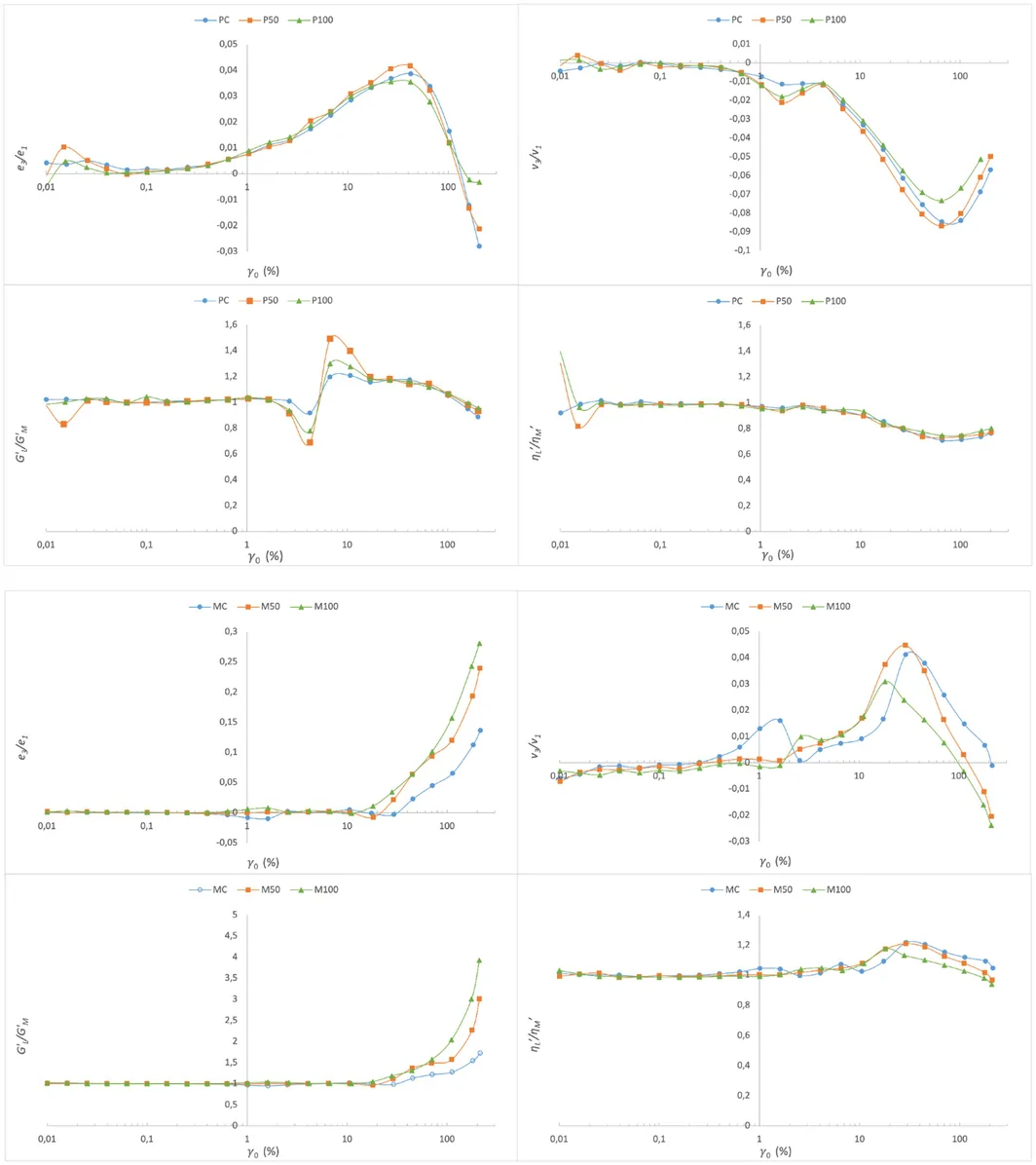
Elastic and viscous LAOS parameters at 6.28 rad/s frequency for pancake samples and meringue samples: *e*_3_/*e*_1_; *v*_3_/*v*_1_; *G*′_L_/*G*′_M_; *η*′_L_/*η*′_M_.

The nonlinear behavior of samples could be better understood with the Chebyshev harmonic analysis. The ratio of the third‐order Chebyshev coefficients (*e*_3_, *v*_3_) to first‐order Chebyshev coefficients (*e*_1,_ *v*_1_) are utilized to explicate the nonlinear properties of samples. The strain softening behavior is indicated by *e*_3_/*e*_1_ < 0, while the strain stiffening behavior is indicated by *e*_3_/*e*_1_ > 0. Additionally, the shear thinning and thickening properties are also defined with the same intervals, according to *v*_3_/*v*_1_, respectively. In the LVR, *e*_3_ */*e_1_ and *v*_3_ */v*_1_ are equal to zero.

As previously indicated, pancake samples showed strain stiffening behavior (*e*_3_/*e*_1_ > 0), while shear thinning behavior (*v*_3_/*v*_1_ < 0) in the nonlinear region. In addition, strain softening behavior was observed by increasing strain amplitude by 200% according to elastic properties. The *e*_3_/*e*_1_ and *v*_3_/*v*_1_ values of meringue samples indicate the strain stiffening and shear thickening in the nonlinear region, respectively. Due to the further deformations of the structure at a higher strain value (200%), sample behavior shifts to shear thinning.

Aquafaba has a lower protein content than egg white and the protein fraction of aquafaba has a lower molecular weight protein molecule than egg white. This points to a potentially lower ability of aquafaba to develop networks than that of egg white (Buhl et al. 2019; Tufaro and Cappa 2023; Tan et al. 2025). However, it is important to note that in the formation of aquafaba foams, not only proteins are involved, but also saponins and soluble carbohydrates. Saponins may also contribute to foam formation due to their ability to decrease surface tension, but they may also cause weaker mechanical interactions compared to proteins (Tufaro and Cappa 2023; Tan et al. 2025). Consequently, when the reductions in the stored and dissipated energies are considered together with the elastic and viscous responses of the aquafaba‐containing samples in the nonlinear region, the results indicate that these products exhibited lower mechanical resistance.

### 3.3 Results of Meringue and Pancake Samples

#### 3.3.1 Texture

Texture profile results of pancake samples obtained by using different amounts of aquafaba as an egg replacer are given in Table 4. The use and ratio of aquafaba as an egg white substitute created a considerable difference in the hardness values of the pancake samples (*p* < 0.05). While the pancake without aquafaba was the hardest sample, in contrast, 100% aquafaba was the softest. Previously, it was stated that the highest batter density was found for the control pancake sample. Consequently, the air bubbles trapped in the batter are increased and a softer product is formed with decreasing batter density. Similar findings were obtained by using aquafaba as an egg replacement in the sponge cake samples (Mustafa et al. 2018). Bird et al. (2017) also reported that the bread made with aquafaba and chickpea soaking water was softer than the control samples. Similar trends were observed for springiness, cohesiveness, gumminess, chewiness, and elasticity values of pancakes. Similar results have been reported in previous studies, indicating that the limited network‐forming ability of plant‐based egg white replacers than egg white may weaken the structure. This weakness may result in reduced springiness and chewiness due to reduced structural integrity (Topaloğlu Günan and Yolci Ömeroğlu 2025).

**TABLE 4:** Texture profile results of pancake and meringue samples.

Pancake samples							Meringue samples			
Sample	Hardness (N)	Springiness	Cohesiveness	Gumminess (N)	Chewiness (N)	Elasticity	Sample	Hardness (N)	Fracturability (N)	Resilience
PC	47.49 ± 0.51^c^	0.95 ± 0.02^b^	0.65 ± 0.04^a^	28.14 ± 2.35^b^	27.80 ± 2.83^a^	0.34 ± 0.02^a^	MC	69.37 ± 2.28^c^	60.09 ± 1.86^c^	0.089 ± 0.006^a^
P50	43.42 ± 0.52^b^	0.93 ± 0.02^b^	0.64 ± 0.02^a^	26.24 ± 1.92^b^	24.11 ± 2.36^a^	0.33 ± 0.03^a^	M50	51.34 ± 1.93^b^	47.67 ± 1.46^b^	0.044 ± 0.003^a^
P100	38.26 ± 2.03^a^	0.8 ± 0.06^a^	0.61 ± 0.05^a^	21.62 ± 1.84^a^	17.98 ± 1.82^b^	0.32 ± 0.01^a^	M100	43.28 ± 1.18^a^	42.68 ± 1.59^a^	0.05 ± 0.003^a^

*Note:* Different letters in the same column (a, b, c) indicate significant differences between means (*p* < 0.05).

Firmness, hardness, and resilience values of meringue samples obtained by using aquafaba as an egg substitute at different rates are given in Table 4. The firmness and hardness of samples decreased with increasing aquafaba amount. In the study conducted by Stantiall et al. (2018), the use of aquafaba in the samples caused a decrease in the hardness value of the meringue. Consistent with our findings, Laursen et al. (2025) reported that increasing the amount of aquafaba compared to egg white protein caused a clear reduction in firmness, highlighting the critical role of egg white proteins in maintaining batter structure. The research also indicates that interfacial competition or structural incompatibilities may arise when egg white protein and plant protein are combined, and that the ratios employed significantly influence whether synergistic or competitive effects dominate.

The decrease in the density values of the products with increasing aquafaba content initially indicates that the samples became lighter and more aerated. However, the lower *G*′ values and the reductions in stored and dissipated energy observed from the Lissajous‐Bowditch curves suggest that the aquafaba‐containing systems exhibited a lower overall stress response and lower resistance to large deformation than the controls. Although all pancake samples showed strain‐stiffening and shear‐thinning behavior and all meringue samples showed strain‐stiffening and shear‐thickening behavior, aquafaba replacement reduced the magnitude of the elastic and dissipative responses without altering the type of nonlinear behavior observed in either product system. These rheological findings were consistent with the reductions in hardness and resilience in the pancake samples and with the lower hardness, fracturability, and resilience values of the aquafaba‐containing meringues. Overall, aquafaba promoted the formation of lighter structures but provided lower mechanical resistance than egg white under large deformation. Similarly, Tan et al. (2025) attributed the lowest hardness, cohesiveness, and springiness values observed in vegan pancakes prepared with aquafaba to the weak structure resulting from differences in protein content.

#### 3.3.2 Color

L*, a*, and b* values expressing the crumb and crust color characteristics of pancake samples are given in Table 5. It was determined that the L* value, which expresses the brightness of the pancake samples, increased in the crumb parts, and decreased in the crust parts as the aquafaba ratio increased (*p* < 0.05). a* value, which expresses the redness of the pancake samples, showed the highest value for the crumb and crust in the sample containing 50% aquafaba. Similarly, according to Mustafa et al. (2018), L* value decreased and a* and b* values increased in the crust parts by using aquafaba as an egg substitute in cake samples. The differences in crust color between both pancakes made with egg white and aquafaba may be due to Maillard and caramelization reactions because of the simple sugar and polysaccharides content of the aquafaba (Shim et al. 2018). Since there was no difference between the crumb and crust colors of the meringue samples, the color analysis was made by powdering the samples. The L* and a*, b* values of the ground and homogenized meringue samples decreased significantly with increasing aquafaba replacement (*p* < 0.05) (Table 5).

**TABLE 5:** Color parameter of pancake and meringue samples.

Pancake samples							Meringue samples			
Sample	Crumb			Crust			Sample			
	L*	a*	b*	L*	a*	b*		L*	a*	b*
PC	64.24 ± 1.92^a^	−0.18 ± 0.04^a^	16.46 ± 0.17^a^	76.28 ± 0.87^c^	3.61 ± 0.35^a^	25.008 ± 0.46^a^	MC	81.41 ± 0.64^a^	6.43 ± 0.13^c^	26.66 ± 0.38^c^
P50	68.93 ± 1.16^b^	0.41 ± 0.08^c^	16.93 ± 0.31^a^	71.96 ± 0.57^a^	6.005 ± 0.93^b^	25.82 ± 1.34^a^	M50	80.91 ± 0.52^a^	5.13 ± 0.1^a^	23.5 ± 0.24^a^
P100	73.09 ± 0.65^c^	0.13 ± 0.001^b^	16.84 ± 0.79^a^	74.83 ± 0.44^b^	3.76 ± 0.29^a^	25.11 ± 0.97^a^	M100	81.02 ± 1.33^a^	5.62 ± 0.33^b^	24.88 ± 0.36^b^

*Note:* Different letters in the same column (a, b, c) indicate significant differences between means (*p* < 0.05).

#### 3.3.3 SEM

The macro and microstructure of pancakes and meringue samples with and without eggs was determined with SEM. The micrographs of pancakes and meringue samples produced by using different ratios of aquafaba with ×1000 magnification are given in Figure 5. When the micrographs of the pancake samples are examined, it is seen that the porosity of the pancake samples decreases with the increase in the aquafaba ratios. It was observed that with the increase in the aquafaba ratio in the meringue formulation, the sample showed a more layered and fragile structure. It is seen that the meringue sample containing 100% aquafaba is more fragile than the other samples. In the study conducted by Paraskevopoulou et al. (2015), it was stated that the porosity of the samples decreased by reducing the egg ratio by using whey isolate in cake samples.

**FIGURE 5:**
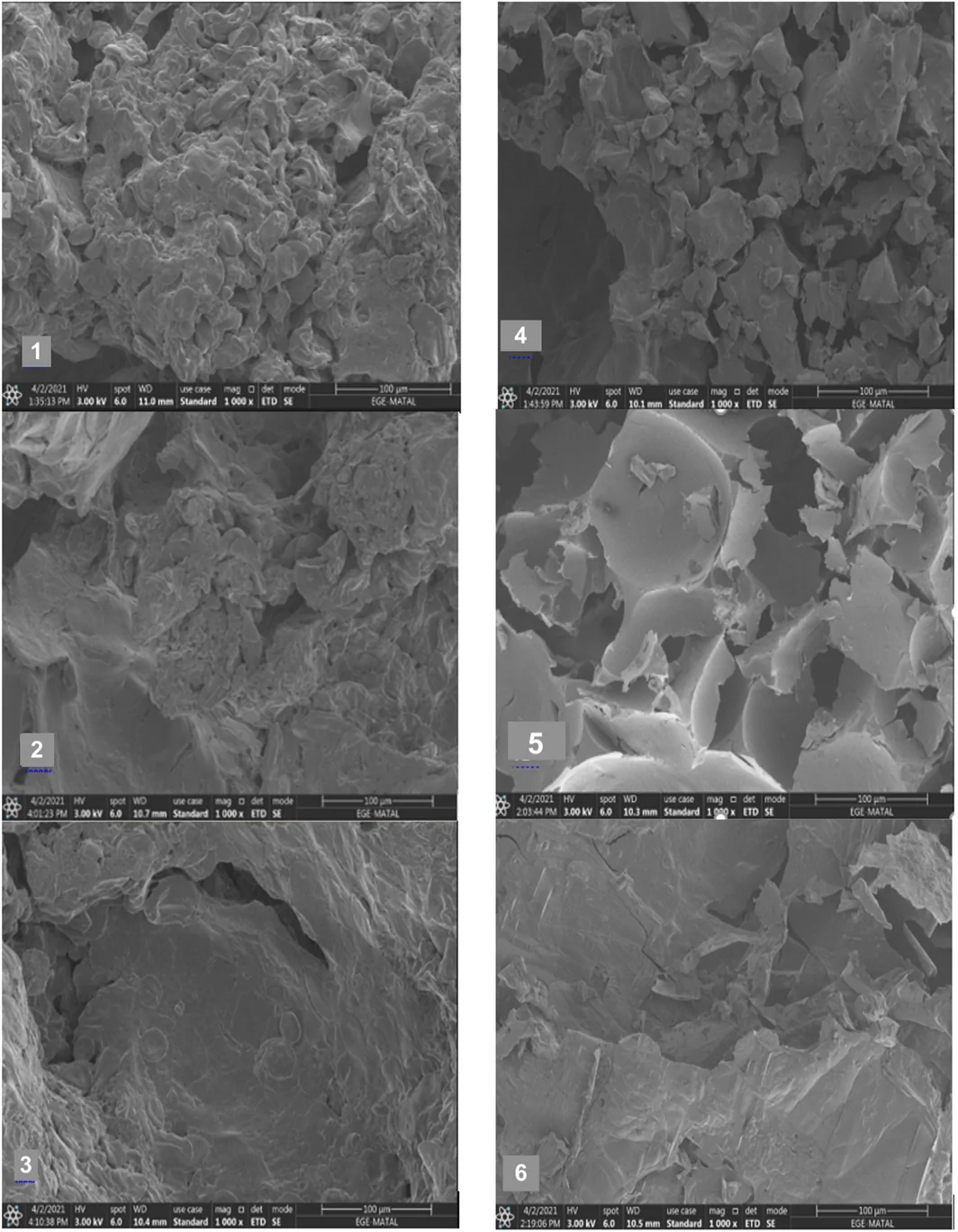
Scanning electron microscope images of pancake and meringue samples (1) PC, (2) P50, (3) P100, (4) MC, (5) M50, (6) M100.

The lower density values were consistent with the formation of lighter pancake and meringue samples, whereas the reductions in stored and dissipated energies, together with the decreases in textural parameter values, suggested that increasing aquafaba replacement was associated with lower resistance to mechanical deformation. In addition, examination of the SEM images indicated that, although well aerated batters were initially obtained, the aquafaba containing samples were less able than the egg white samples to retain these structures during baking and could, therefore, be considered less resistant to compression and fracture.

In addition to the SEM images, photographs of the samples are presented in Figure 6. In the pancake samples, the use of aquafaba as an egg replacer resulted in a reduction in the number of pores. Similarly, the images of the meringue samples showed structural changes as the replacement level increased, together with a decrease in the number of visible pores. These images support the previous findings by indicating that, although the pancake and meringue samples were initially well aerated and exhibited lower density values, they were unable to retain this structure effectively during baking. This observation was consistent with their lower nonlinear mechanical resistance and reduced textural strength.

**FIGURE 6:**
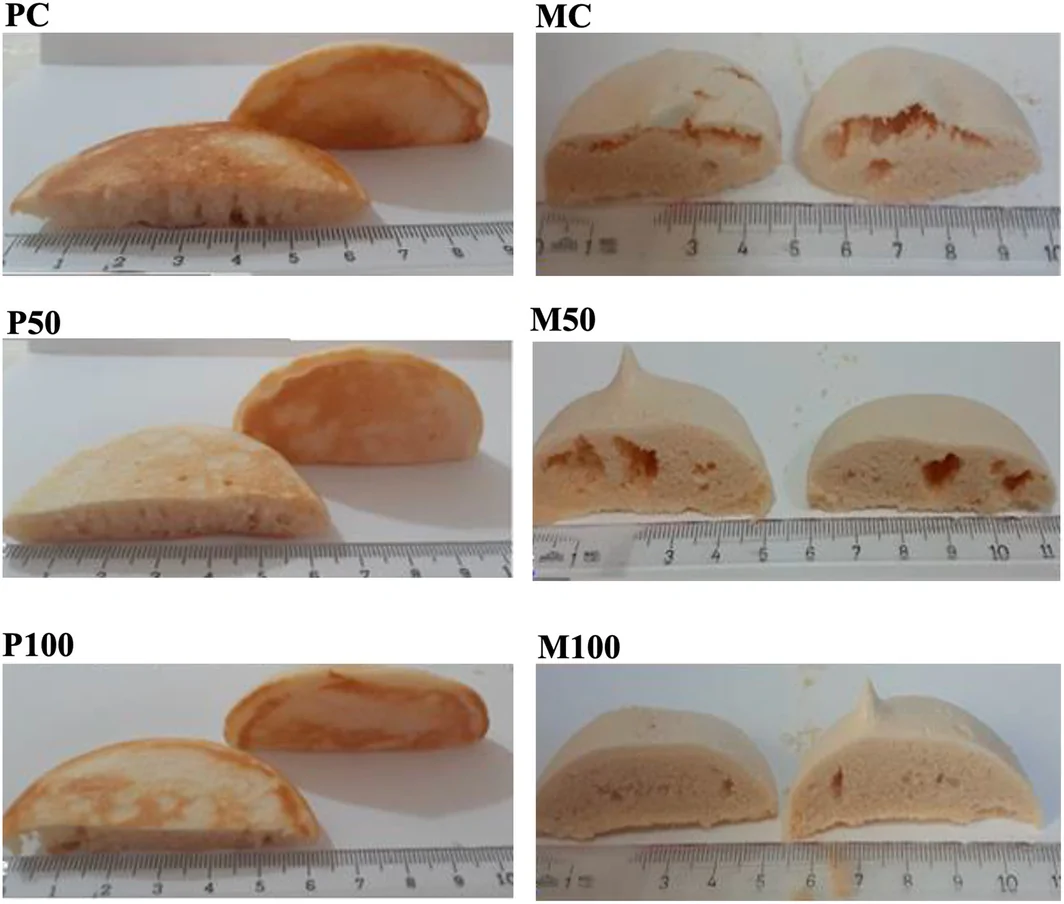
Images of pancake and meringue samples.

## 4 Conclusion

This study was undertaken to use aquafaba as a food ingredient instead of being disposed of as waste and to evaluate the effect of aquafaba on pancake and meringue samples as an egg white substitute. Pancake and meringue samples containing purely herbal egg substitutes (aquafaba) and suitable for the consumption of individuals who do not consume eggs due to egg allergy and vegan diet preferences were produced. The addition of aquafaba significantly influenced the nonlinear rheological behavior of both pancake batter and meringue systems due to its composition of soluble proteins, surface‐active saponins, and polysaccharides. The Lissajous curves revealed that the elastic and viscous parts of meringue and pancake batter samples were highly strain dependent. As the aquafaba substitution increased, the dissipated and stored energies of both the pancake and meringue samples decreased. In the nonlinear region, pancake and meringue samples showed strain stiffening behavior (*e*_3_/*e*_1_ > 0) according to elastic properties. In addition, shear thinning behavior (*v*_3_/*v*_1_ < 0) and shear thickening (*v*_3_/*v*_1_ > 0) behavior were observed for the pancake and meringue samples in the nonlinear region, respectively. As the aquafaba substitution ratio increased, the hardness values decreased, making the pancakes and meringue softer. Overall, the decrease in density with increasing aquafaba replacement suggested a greater initial incorporation of air in the pancake batters and meringue foams. However, the decreases in stored and dissipated energies, together with the lower *G*′ values, suggested that the aquafaba‐containing samples were less resistant to large deformation. The SEM and cross‐sectional images indicated less effective retention of the incorporated air during baking. The research has shown that the use of aquafaba as an egg white substitute can have similar functional properties to eggs, and it is a suitable and inexpensive alternative for use in meringue and pancake samples. In addition, a new study has been contributed to the studies on the characterization of nonlinear rheological properties of batter and foam prepared with aquafaba.

Future research should investigate the variation in protein, saponin, starch, and soluble carbohydrate contents between chickpea cultivars and genotypes and whether this influences the LAOS behavior and structural performance of aquafaba. Such studies may provide information on compositional features related to improved air incorporation, deformation resistance, and structural retention during processing. The LAOS analysis could also be used for other high‐shear plant‐based foods, such as vegan mayonnaise and aerated creams, to help develop more stable egg‐free formulations. Finally, sensory assessment, consumer acceptance, and shelf‐life studies are necessary to validate the practical application of the most promising pancake and meringue formulations.

## Author Contributions

**Selen Selen Erkoç:** data curation, formal analysis, investigation, writing – original draft, visualization. **Esra Özyiğit:** data curation, investigation, formal analysis, writing – review and editing, visualization. **İpek Özcan:** writing – original draft, investigation, formal analysis. **Seher Kumcuoglu:** supervision, project administration, writing – review and editing, conceptualization. **Şebnem Tavman:** supervision, project administration, conceptualization, writing – review and editing.

## Funding

This study was financially supported by Ege University, Scientific Research Project (BAP) Coordination Unit (Project No: FGA 2020‐22346).

## Disclosure

All authors have read and approved the manuscript; and all co‐authors are aware of its submission to the Food Science and Nutrition.

## Ethics Statement

We affirm adherence to scientific ethical principles and guidelines in this research article.

## Consent

The authors have nothing to report.

## Conflicts of Interest

The authors declare no conflicts of interest.

## Data Availability

The data that support the findings of this study are available from the corresponding author upon reasonable request.
